# A novel risk score system of immune genes associated with prognosis in endometrial cancer

**DOI:** 10.1186/s12935-020-01317-5

**Published:** 2020-06-15

**Authors:** Hongyu Zhou, Chufan Zhang, Haoran Li, Lihua Chen, Xi Cheng

**Affiliations:** 1 Department of Gynecological Oncology, Fudan University Shanghai Cancer Center, 270 Dong’an Road, Shanghai, 200032 China; 2 Department of Medical Oncology, Fudan University Shanghai Cancer Center, Shanghai, China; 3 Department of Oncology, Shanghai Medical College, Fudan University, Shanghai, China; 4 Cancer Institute, Fudan University Shanghai Cancer Center, Shanghai, China

**Keywords:** The Cancer Genome Atlas (TCGA) database, Endometrial cancer, Differentially expressed genes, Risk score, Prognosis

## Abstract

**Background:**

Endometrial cancer was the commonest gynecological malignancy in developed countries. Despite striking advances in multimodality management, however, for patients in advanced stage, targeted therapy still remained a challenge. Our study aimed to investigate new biomarkers for endometrial cancer and establish a novel risk score system of immune genes in endometrial cancer.

**Methods:**

The clinicopathological characteristics and gene expression data were downloaded from The Cancer Genome Atlas (TCGA) database. Differentially expressed genes (DEGs) of immune genes between tumors and normal tissues were identified. Protein–protein interaction (PPI) network of immune genes and transcriptional factors was integrated and visualized in Cytoscape. Univariate and multivariate analysis were employed for key genes to establish a new risk score system. Receiver operating characteristic (ROC) curve and survival analysis were performed to investigate the prognostic value of the model. Association between clinical characteristics and the model was analyzed by logistic regression. For validation, we identified 34 patients with endometrial cancer from Fudan University Shanghai Cancer Center (FUSCC). We detected 14-genes mRNA expression and calculated the risk scores of each patients and we performed survival analysis between the high-risk group and the low-risk group.

**Results:**

23 normal tissues and 552 tumor tissues were obtained from TCGA database. 410 immune-related DEGs was identified by difference analysis and correlation analysis. KEGG and GO analysis revealed these DEGs were enriched in cell adhesion, chemotaxis, MAPK pathways and PI3K-Akt signaling pathway, which might regulate tumor progression and migration. All genes were screened for risk model construction and 14 hub immune-related genes (HTR3E, CBLC, TNF, PSMC4, TRAV30, PDIA3, FGF8, PDGFRA, ESRRA, SBDS, CRHR1, LTA, NR2F1, TNFRSF18) were prognostic in endometrial cancer. The area under the curve (AUC) was 0.787 and the high-risk group estimated by the model possessed worse outcome (P < 0.001). Multivariate analysis suggested that the model was indeed an independent prognostic factor (high-risk vs. low-risk, HR = 1.14, P < 0.001). Meanwhile, the high-risk group was prone to have higher grade (P = 0.002) and advanced clinical stage (P = 0.018). In FUSCC validation set, the high-risk group had worse survival than the low-risk group (P < 0.001).

**Conclusions:**

In conclusion, the novel risk model of immune genes had some merits in predicting the prognosis of endometrial cancer and had strong correlation with clinical outcomes. Furthermore, it might provide new biomarkers for targeted therapy in endometrial cancer.

## Background

Endometrial cancer was the leading gynecological malignancy in western countries and ranked sixth in women cancer worldwide [1, 2]. There were estimated 63,230 new cases and 11,350 deaths in female uterine corpus carcinoma in 2018 [3] and the incidence was still rising [4, 5]. Most newly diagnosed patients had favorable prognosis due to the early stage with 5-year survival rate over 80% [6]. However, patients with delayed diagnosis were proved to have dismal survival [7].

Uteri corpus endometrial carcinoma (UCEC) was often classified into two histological type: type I (estrogen-dependent endometrioid adenocarcinomas) and type II (estrogen-independent serous carcinomas). Type I carcinomas represented 80% of endometrial cancer with favorable prognosis and good response to estrogen treatment. Conversely, type II tumors, insensitive to estrogen, only accounting for 10–20%, had extremely aggressive behavior as advanced stage, distant metastasis [8].

Multimodality strategies, surgery followed by adjuvant therapy, had achieved great success in early-staged patients. Nevertheless, the treatment and management of advanced stage and recurrent patients still remained a challenge. Pre-clinical and clinical investigations of targeted therapies suggested efficacy for some agents. Single agent targeted therapies, however, had modest activity. Identifying potential biomarkers that effectively responded to targeted therapy appeared extremely urgent.

There were rarely specific gene alterations investigated in endometrial cancer. Mismatch repair genes (typically MLH1, MSH2, PMS2, or MSH6) mutation had been reported in Lynch syndrome related endometrial cancer [7]. BAF250a expression, also known as ARID1A, was commonly detected absent in high-grade endometrioid endometrial cancer at about 40% [9]. CTNNB1, commonly mutated in low grade, early stage endometrial cancer, were associated with worse recurrence-free survival [10]. FGFR2 mutation was also reported mutated in endometrial cancer [11]. Human epidermal growth factor receptor 2 (HER2)/neu, a receptor was discovered overexpressed in 30% of uterine serous carcinoma [12]. P53 protein, if absent or diffusely overexpressed, was associated with poor prognosis [8].

Recently, TCGA research group performed an integrating genomic characterization of endometrial cancer by whole-exome sequence analysis and proposed to divide it into four subgroups as “POLE ultra-mutated”, “hypermutated/microsatellite unstable”, “copy number”, “low/microsatellite stable” and “copy number high (serous-like)” [13–15]. Above this, in our study, we were devoted to exploring new biomarkers and establishing a risk score model to predict prognosis, aiming to provide novel therapeutic options of personalized medicine for endometrial cancer.

## Methods

### Data collection and enrichment analysis

Total 23 normal cases and 552 tumor cases with clinicopathological characteristics and expression data were downloaded from TCGA official website for the Uterine Corpus Endometrial Carcinoma projects (UCEC). 2498 immune-related genes data was downloaded from IMMPORT website. 410 immune genes were verified with |Log Fc| ≥ 2 and FDR < 0.25 by differential analysis. Gene Ontology (GO) analysis of these DEGs between UCEC and normal tissues was performed using blast2GO with P-value ≤ 1 and pathway enrichment analysis was carried out against the KEGG database with Q-value ≤ 1.

### PPI network and survival analysis

The data of transcriptional factors (TFs) was obtained from Cistrome database. Differential expressed TFs were identified by the criteria of |Log FC| ≥ 1 and P value < 0.05. PPIs with a confidence score ≥ 0.4 and P value < 0.001 were reserved and further visualized in Cytoscape.

For survival analysis, we deleted partial cases with incomplete follow-up information, finally, 544 patients were reserved for further analysis. Univariate analysis was conducted to identify candidate genes which were significantly correlated with survival with P value < 0.05. Multivariate Cox hazards regression model was established to select independent prognostic genes. We constructed a prognostic gene signature according to a linear combination of gene expression values multiplied by a regression coefficient (β) accessed from the multivariate Cox proportional hazards regression model of each gene. The formula is as follows: risk score = expression of gene1 × β_1_gene_1_ + expression of gene_2_ × β_2_gene_2_ +… expression of gene_n_ × β_n_gene_n_ [16, 17]. All patients were divided into low- or high-risk groups according to the median risk score. Receiver operating characteristic (ROC) curve analysis by R package was performed to assess the predictive accuracy of the prognostic value for time-dependent cancer death.

The area under the curve (AUC) was calculated to measure the predictive ability of the gene signature for clinical outcomes [18].

### Validation of the model

We obtained 34 patients diagnosed with endometrial cancer from Fudan University Shanghai Cancer Center (FUSCC) between January 2017 and February 2018. RNAs were distracted from total 34 tumor samples and cDNAs of these samples were synthesized by reverse transcription reaction kit (Takara, RR036A). Then we performed real time polymerase chain reaction (RT-PCR) (Takara, RR820A) to estimate 14 genes mRNA expression level in endometrial cancer. The internal reference was GAPDH mRNA. The primer sequences for 14 genes mRNA and GAPDH mRNA were listed in Additional file 1: Table S1. We calculated risk scores of each patients according to the 14 genes mRNA expression level observed to the formula of the model, and then we divided all 34 patients into the two groups: the high-risk group and the low-risk group according to the median value of risk scores. Survival analysis was performed in the two groups.

### Statistical analysis

All statistical analysis were conducted by software R (v.3.6.1). The association between clinicopathologic characteristics and risk score system was analyzed by Wilcoxon signed-rank test and logistic regression. Kaplan–Meier method and Cox multivariate model were used for survival analysis. The cut-off value of risk score was determined by the median value.

## Results

### Identification of immune-related DEGs and functional annotation

**Fig. 1:**
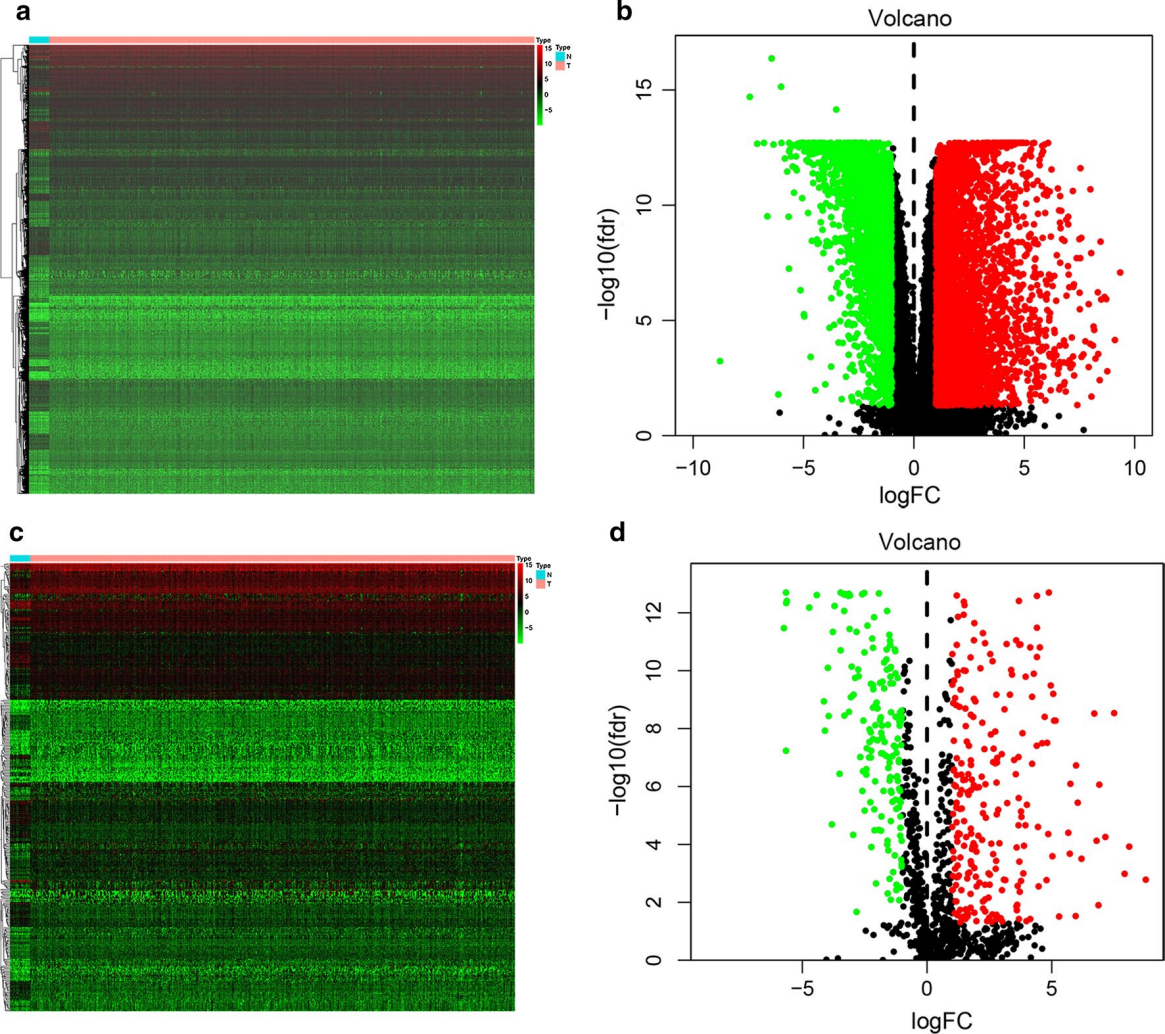
Identification of DEGs. **a**, **b** Heatmap and volcano plots of 6268 DEGs in endometrial cancer and normal tissues from TCGA database. **c**, **d** Heatmap and volcano plots of 410 immune-related DEGs. The colors in the heatmaps from green to red represent expression level from low to high. The red dots in the volcano plots represent up-regulation, the green dots represent down-regulation and black dots represent genes without differential expression

Gene expression data was downloaded from TCGA database. 23 normal tissues and 552 tumor tissues were obtained and 6268 DEGs by difference analysis were shown in heatmap and volcano plot (in Fig. 1a, b). To identify immune-related genes of DEGs, 2498 immune genes data was downloaded from IMMPORT website. Eventually, 410 immune-related DEGs were obtained (shown in Fig. 1c, d, in Additional file 1: Table S2).

**Fig. 2:**
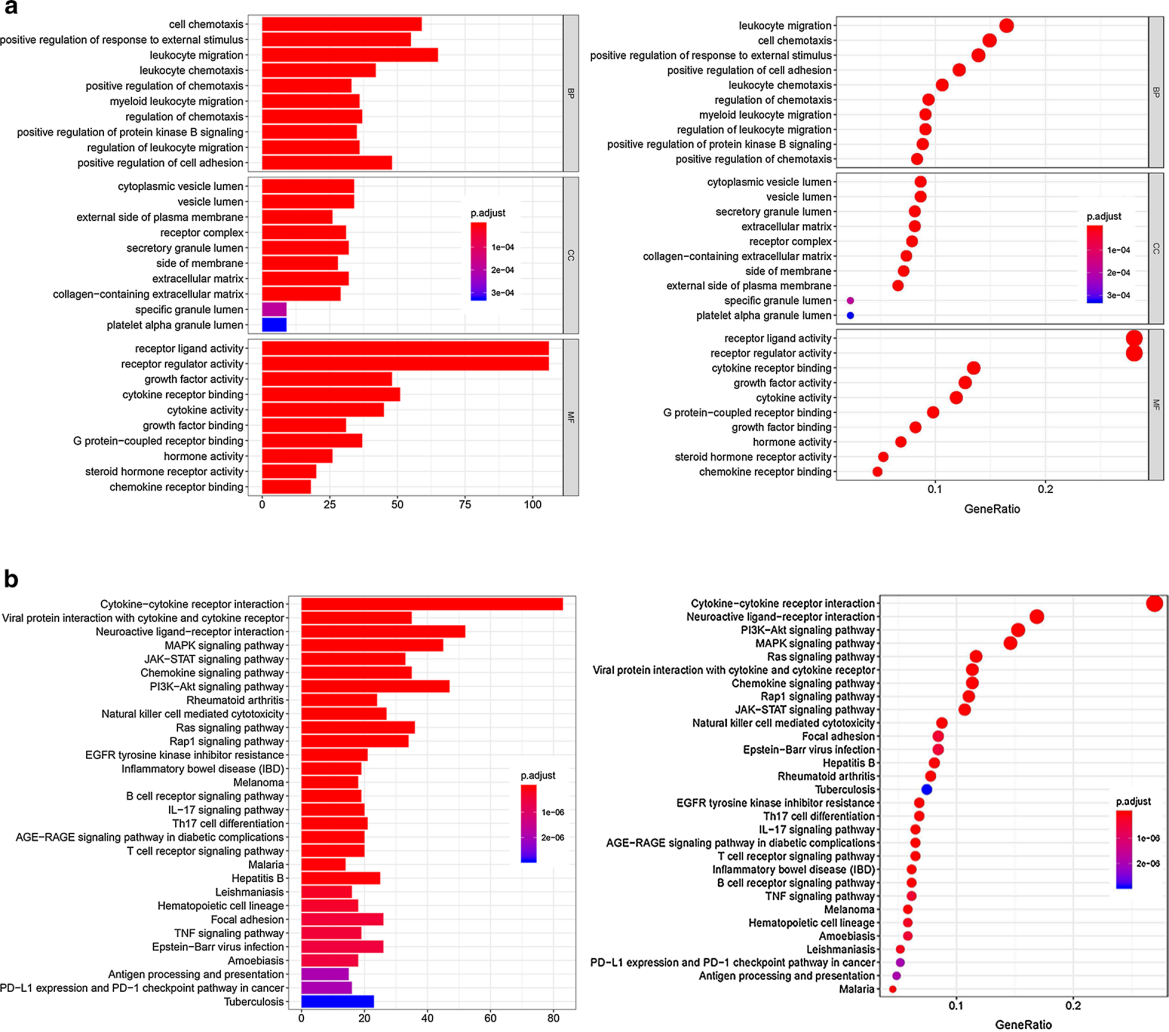
GO and KEGG enrichment analysis of DEGs. **a**, **b** GO analysis. GO analysis divided DEGs into three functional groups: molecular function (MF), biological processes (BP), and cell composition (CC). **c**, **d** KEGG analysis of DEGs

Moreover, we performed KEGG and GO enrichment analysis to elucidate the functional role of DEGs. The results of GO analysis revealed that “positive regulation of cell adhesion”, “positive regulation of protein kinase B signaling”, and “positive regulation of chemotaxis”, “regulation of leukocyte migration” were significantly enriched biological processes and pathways, which might be associated with tumor migration and tumor microenvironment (in Fig. 2a, b). KEGG enrichment analysis manifested that these genes were probably enriched in cytokine–cytokine receptor interaction, PI3K-Akt signaling pathway, MAPK signaling pathway and Ras signaling pathway which might regulate cancer progression (in Fig. 2c, d).

### Interaction network for immune-specific genes and transcription factors

**Fig. 3:**
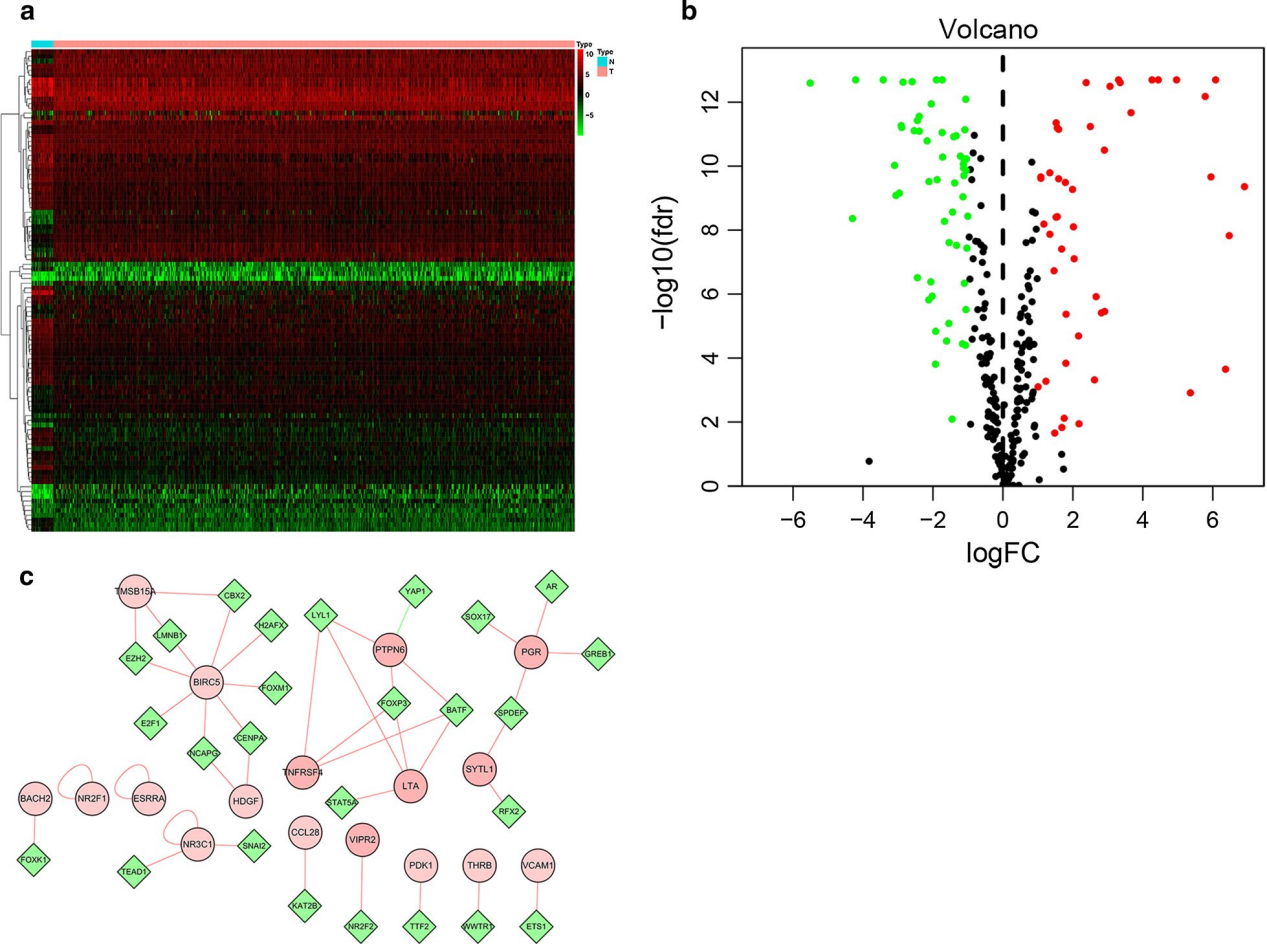
Interaction network for immune-specific genes and transcription factors. **a**, **b** Heat map and Volcano plots of differentially expressed transcription factors (TFs). The colors in the heatmaps from green to red represent expression level from low to high. The red dots in the volcano plots represent up-regulation, the green dots represent down-regulation and black dots represent TFs without differential expression. **c** A significant module from protein–protein interaction network

To find out the transcriptional factors (TFs) for immune-related genes, information on TFs were downloaded from Cistrome database. We investigated differentially expressed TFs with Log FC ≥ 2 and FDR < 0.25 (shown in Fig. 3a, b) and selected TFs relative to immune genes by correlation analysis with the filter for correlation coefficient = 0.04 and P value = 0.001 (data was shown in Additional file 2: Table S2). Integration of protein–protein interaction (PPI) networks was visualized in software Cytoscape (in Fig. 3c).

### Clinical characteristics in TCGA database and FUSCC

Baseline characteristics were downloaded from TCGA database. Patients with incomplete follow-up information were deleted and total 544 patients were identified. In this cohort, shown in Table 1, the median overall survival time was 28 months (1–229 months). Age at diagnosis ranged from 31 to 90 years with median was 64 years. Majority of patients (n = 407, 74.82%) were endometrioid endometrial adenocarcinoma, followed by serous endometrial adenocarcinoma (n = 115, 21.14%) and mixed type was least (n = 22, 4.04%). 40.63% were low/moderate and 59.38% were high grade. 428 (84.42%) patients were founded tumor free but 79 (15.58%) were with tumor. As for lymph node, only 16.74% (n = 74) possessed positive pelvic lymph node and 10.30% were founded positive para-aortic lymph node. Stage I accounted for 62.32% (n = 339), stage II was 9.56% (n = 52) and stage III was 22.61% (n = 123) and stage IV was 5.51% (n = 30). For validation set, we obtained 34 patients with endometrial cancer from FUSCC. Clinicopathological characteristics of these patients were shown in Additional file 3: Table S3.

**Table 1:** Clinical characteristics of 544 patients with UCEC from TCGA database

Clinicopathological characteristics	Number (total = 544) (%)	
Age at diagnosis	31–90 years (median: 64 years)	
Race		
White	372	72.37%
Black or African American	109	21.21%
Other	33	6.42%
Menopause status		
Pre	52	10.46%
Post	445	89.54%
Surgical approach		
Minimally invasive	203	38.96%
Open	318	61.04%
Histological type		
Serous endometrial adenocarcinoma	115	21.14%
Endometrioid endometrial adenocarcinoma	407	74.82%
Mixed serous and endometrioid	22	4.04%
Grade		
Low/moderate (G1/G2)	221	40.63%
High (G3)	323	59.38%
Tumor invasion depth		
< 1/2	254	55.70%
≥ 1/2	202	44.30%
Tumor status		
Tumor free	428	84.42%
With tumor	79	15.58%
Residual tumor		
No residual (R0)	375	90.80%
With residual (R1/R2)	38	9.20%
Peritoneal washing		
Negative	352	85.85%
Positive	58	14.15%
Pelvic lymph node		
Negative	368	83.26%
Positive	74	16.74%
Para-aortic lymph node		
Negative	331	89.70%
Positive	38	10.30%
Stage		
I	339	62.32%
II	52	9.56%
III	123	22.61%
IV	30	5.51%

### Construction of risk score system

**Fig. 4:**
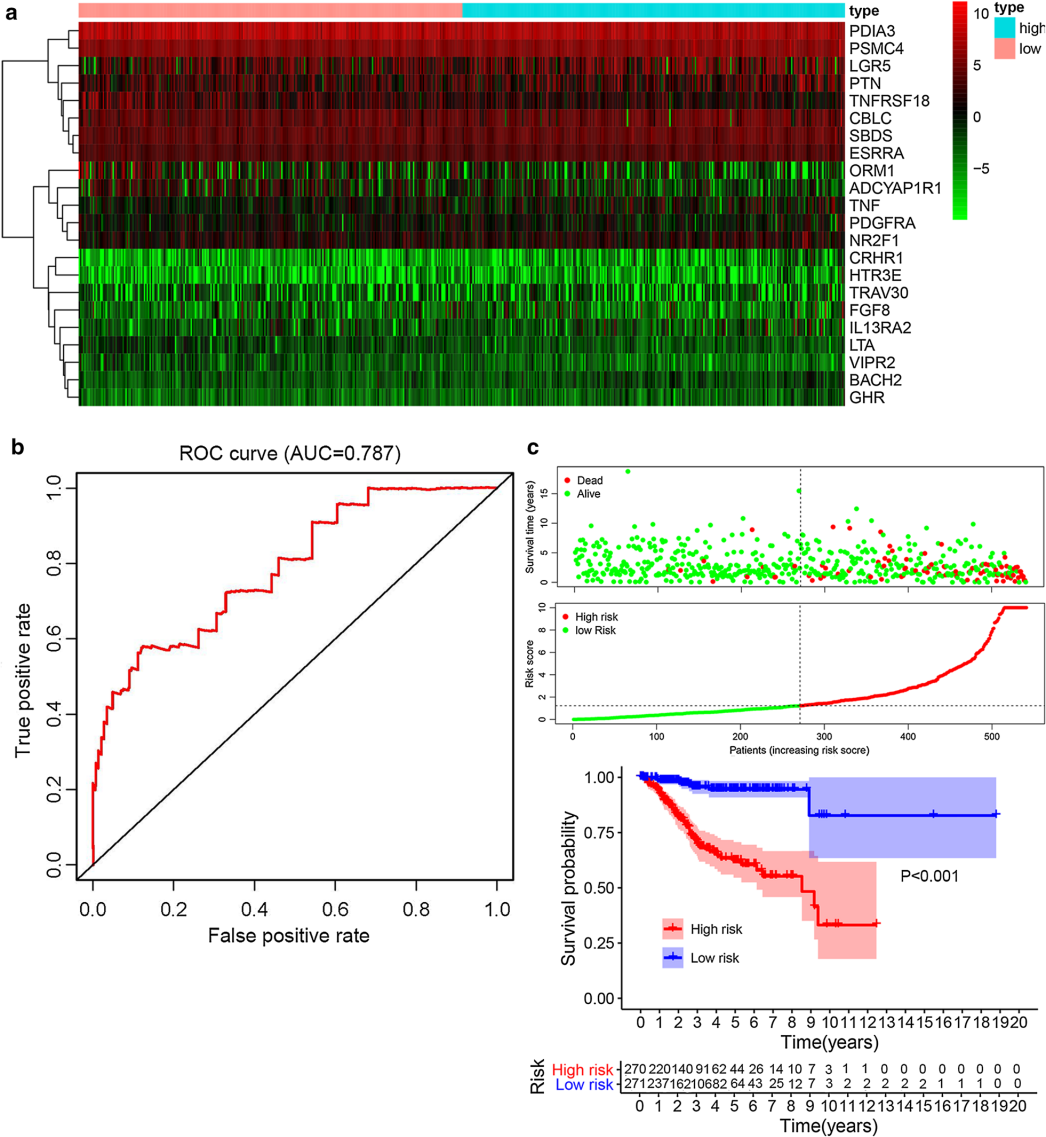
Construction of risk score system. **a** Risk score estimated of each patient on the basis of 14 hub genes expression. **b** ROC curve for the risk model. **c** Patients were divided into high risk group and low risk group. Survival analysis of the patients is also shown

Univariate and multivariate survival analysis by Cox proportional hazards models were conducted to select prognostic key genes. All the genes with significant P values were screened for risk model construction. And the risk score algorithm comprised of 14 hub genes (HTR3E, CBLC, TNF, PSMC4, TRAV30, PDIA3, FGF8, PDGFRA, ESRRA, SBDS, CRHR1, LTA, NR2F1, TNFRSF18) was established in Table 2. Otherwise, other 8 genes in this model including VIPR2, LGR5, IL13RA2, PTN, BACH2, GHR, ADCYAP1R1, ORM1 were not significant with overall survival. Risk scores could be calculated as: HTR3E * 0.513 + CBLC * 0.015 + TNF * 0.034 + PSMC4 * 0.004 + TRAV30 * 0.110 − PDIA3 * 0.004 + FGF8 * 0.020 + PDGFRA * 0.029 + ESRRA * 0.048 + SBDS * 0.016 + CRHR1 * 0.162− LTA * 0.819 + NR2F1 * 0.019 − TNFRSF18 * 0.028. In this gene model, three of them (PDIA3, LTA, TNFRSF18) were negatively correlated with survival and other 11 genes were positively related to overall survival. The contribution of each gene made to this risk score model was weighted by the value of coefficients. The risk score for each patient was estimated according to the expressions of the 14 hub genes (shown in Fig. 4a).

**Table 2:** Prognostic risk model for endometrial cancer

Name	Coef	HR; 95% CI	P value
HTR3E	0.513	1.67 (1.34–2.09)	0.000
CBLC	0.015	1.02 (1.01–1.02)	0.000
TNF	0.034	1.03 (1.01–1.05)	0.000
PSMC4	0.004	1.00 (1.00–1.01)	0.003
TRAV30	0.110	1.12 (1.04–1.20)	0.004
PDIA3	− 0.004	1.00 (0.99–1.00)	0.004
FGF8	0.020	1.02 (1.01–1.03)	0.006
PDGFRA	0.029	1.03 (1.01–1.05)	0.010
ESRRA	0.048	1.05 (1.01–1.09)	0.013
SBDS	0.016	1.02 (1.00–1.03)	0.016
CRHR1	0.162	1.18 (1.02–1.35)	0.021
LTA	− 0.819	0.44 (0.21–0.93)	0.031
NR2F1	0.019	1.02 (1.00–1.04)	0.032
TNFRSF18	− 0.028	0.97 (0.95–1.00)	0.038
VIPR2	− 1.231	0.29 (0.08–1.09)	0.068
LGR5	0.007	1.01 (1.00–1.01)	0.068
IL13RA2	0.024	1.02 (1.00–1.05)	0.071
PTN	0.005	1.01 (1.00–1.01)	0.080
BACH2	− 0.401	0.67 (0.42–1.07)	0.090
GHR	0.816	2.26 (0.86–5.96)	0.099
ADCYAP1R1	− 0.047	0.95 (0.90–1.01)	0.133
ORM1	− 0.027	0.97 (0.94–1.01)	0.148

*Coef* coefficients, *HR* hazards ratio, *CI* confidence interval

All patients scored by the model could be classified into two groups: the high-risk group and low-risk group (in Fig. 4c). Receiver operating characteristics (ROC) curve was constructed to assess the predictive accuracy and the area under the curve (AUC) was 0.787 (in Fig. 4b). Similarly, results of survival analysis revealed that the high-risk group was associated with worse outcome than the low-risk group (P < 0.001).

### Prognostic value of the risk model and clinical association

**Fig. 5:**
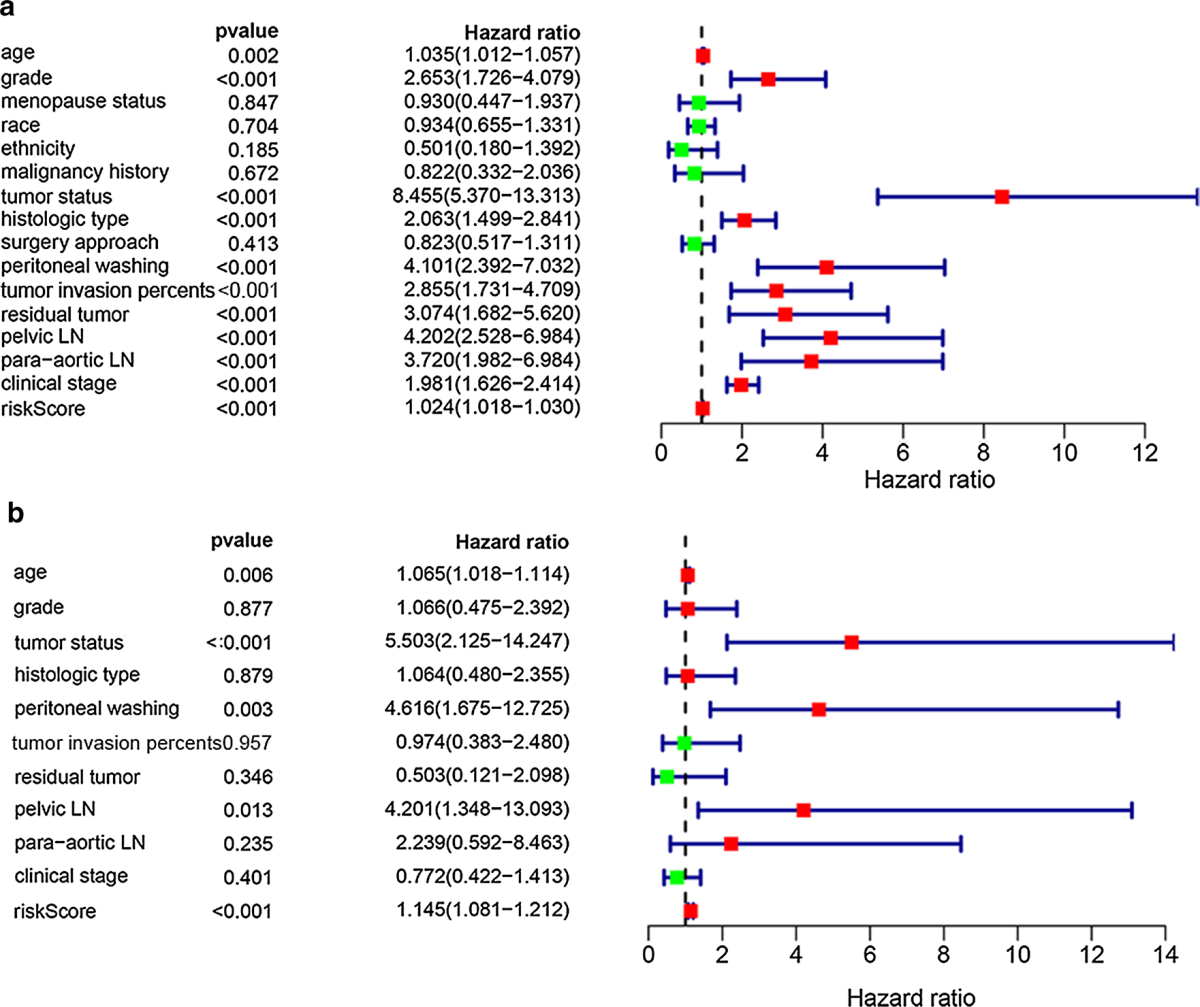
Validation of the prognostic value of the risk model. **a** Univariate regression model. **b** Multi-cox hazards regression model

To validate the prognostic value of this risk model, survival analysis was performed by univariate and multi-cox hazards regression model (in Fig. 5a, b). In Table 3, we discovered that age at diagnosis (≥ 34 year vs. < 34 years, HR = 1.06, P = 0.006), tumor status (with tumor vs tumor free, HR = 5.50, P < 0.001), peritoneal cytology (positive vs. negative, HR = 4.62, P = 0.003), pelvic lymph node (positive vs. negative, HR = 4.20, P = 0.013) and risk model (high-risk vs. low-risk, HR = 1.14, P < 0.001) were independent prognostic factors by multivariate analysis.

**Table 3:** Univariate and multivariate analysis of risk factors for endometrial cancer

Characteristics	HR; 95% CI	P value
Univariate analysis		
Age	1.03 (1.01–1.06)	0.002
Grade	2.65 (1.73–4.08)	0.000
Menopause status	0.93 (0.45–1.94)	0.847
Race	0.93 (0.65–1.33)	0.704
Tumor status	8.45 (5.37–13.31)	0.000
Histologic type	2.06 (1.50–2.84)	0.000
Surgery approach	0.82 (0.52–1.31)	0.413
Peritoneal washing	4.10 (2.39–7.03)	0.000
Tumor invasion percent	2.86 (1.73–4.71)	0.000
Residual tumor	3.07 (1.68–5.62)	0.000
Pelvic LN	4.20 (2.53–6.98)	0.000
Para-aortic LN	3.72 (1.98–6.98)	0.000
Clinical stage	1.98 (1.63–2.41)	0.000
Risk score	1.02 (1.02–1.03)	0.000
Multivariate analysis		
Age	1.06 (1.02–1.11)	0.006
Grade	1.07 (0.48–2.39)	0.877
Tumor status	5.50 (2.13–14.25)	0.000
Histologic type	1.06 (0.48–2.36)	0.879
Peritoneal washing	4.62 (1.67–12.73)	0.003
Tumor invasion percent	0.97 (0.38–2.48)	0.957
Residual tumor	0.50 (0.12–2.10)	0.346
Pelvic LN	4.20 (1.35–13.09)	0.013
Para-aortic LN	2.24 (0.59–8.46)	0.235
Clinical stage	0.77 (0.42–1.41)	0.401
Risk score	1.14 (1.08–1.21)	0.000

*HR* hazards ratio, *CI* confidence interval

**Fig. 6:**
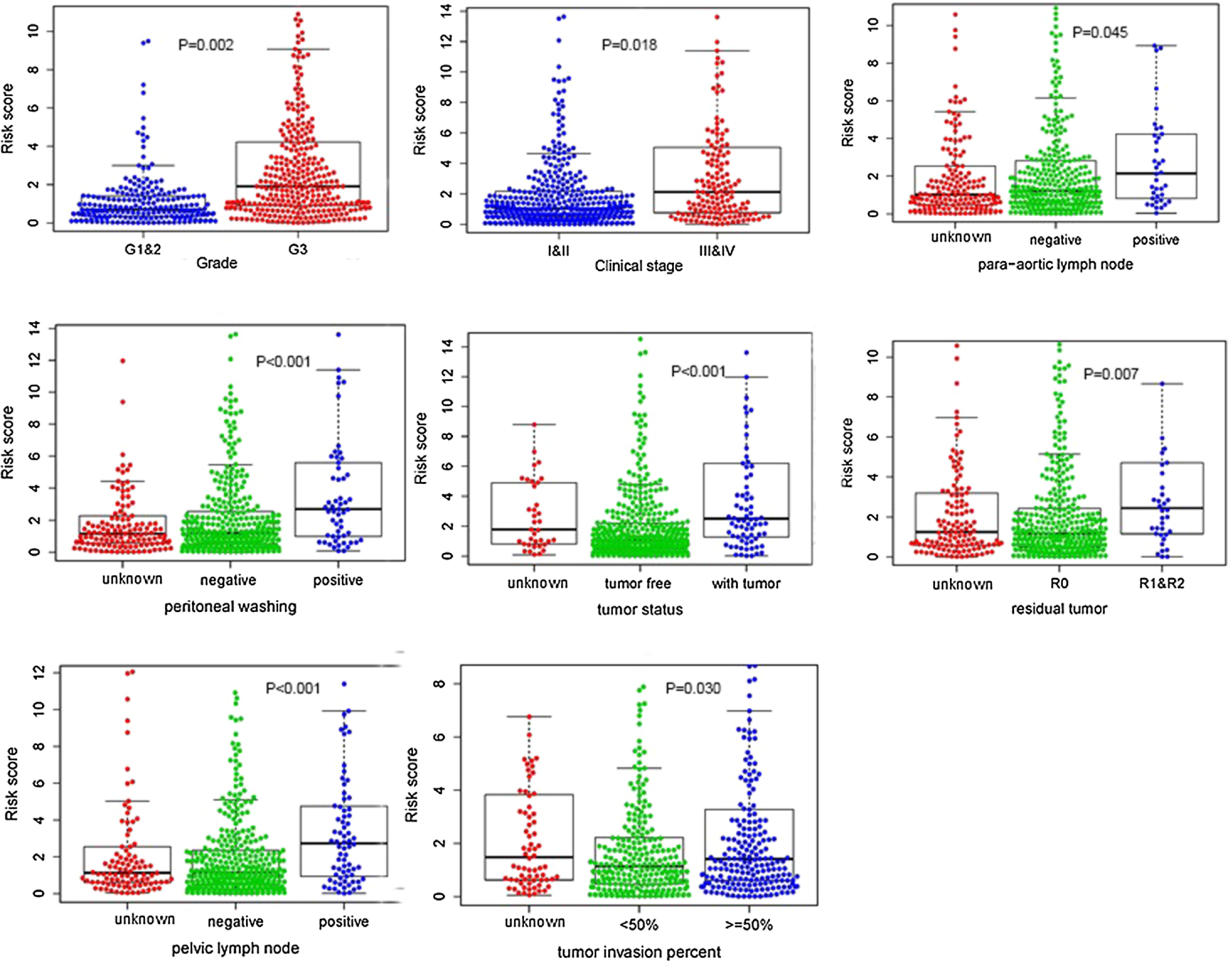
Association between the risk model and different clinical characteristics

Furthermore, we also analyzed the association between risk model and clinical characteristics by logistic regression, and figured out that the high-risk group was prone to possess higher grade (P = 0.002) and advanced clinical stage (P = 0.018) in Fig. 6. Meanwhile, we investigated the correlation between each gene in this model and clinicopathological features separately (in Additional file 4: Figure S1).

## External validation of the model in FUSCC

**Fig. 7:**
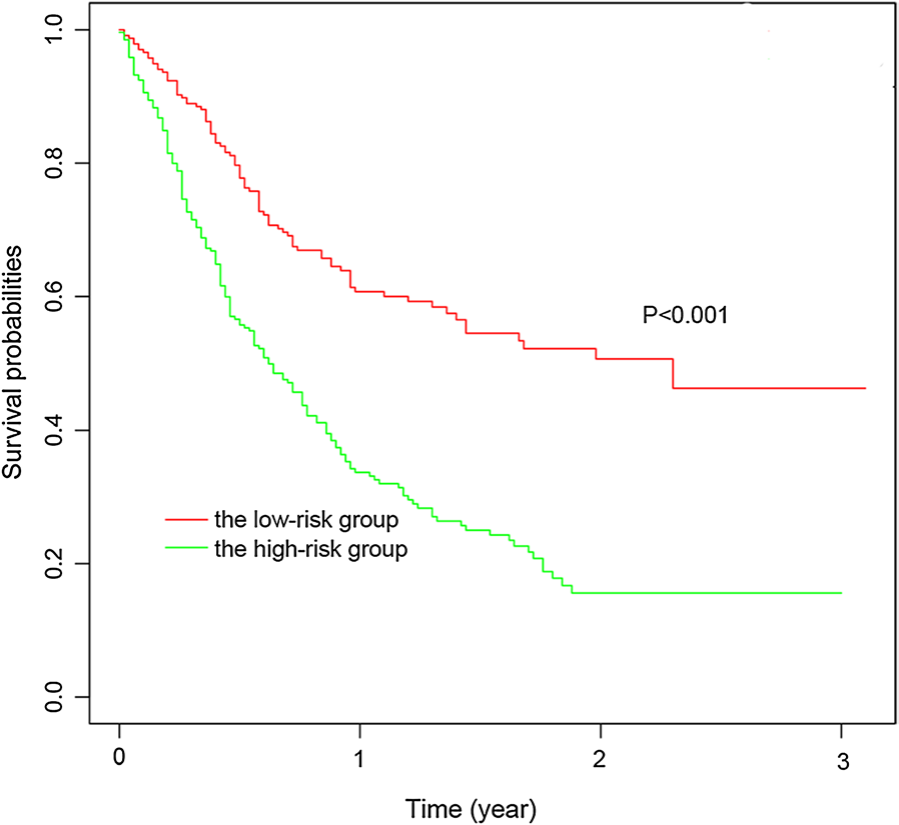
Survival analysis of the high-risk group and the low-risk group scored by the model in FUSCC validation set. All 34 patients were divided into two groups by the median value of risk scores: the high-risk group and the low-risk group

We detected 14-genes mRNAs expression of 34 patients with endometrial cancer in FUSCC and calculated risk scores of each patients according to the formula of the model. All patients were classified into two groups: the high-risk group and the low-risk group by the median value of risk scores. We investigated that the high-risk group had worse survival than the low-risk group significantly with P < 0.001 in Fig. 7.

## Discussion

In our study, we identified and constructed a 14 hub genes risk score model for endometrial cancer. All gene expression data and patients clinical characteristics information were downloaded from TCGA dataset. We analyzed the 6268 DEGs between endometrial cancer and normal tissues and integrated the immune genes from IMMPORT database, eventually, we verified 410 immune-related DEGs. Moreover, univariate and multi-cox regression were employed for the key genes, subsequently, a 14 hub-genes model including HTR3E, CBLC, TNF, PSMC4, TRAV30, PDIA3, FGF8, PDGFRA, ESRRA, SBDS, CRHR1, LTA, NR2F1, TNFRSF18, was successfully established. Furthermore, to investigate the prognostic value of the model, we performed the ROC curve and investigate the association between the model and clinical features. As expected, the high-risk group was correlated with worse overall survival and was inclined to have advanced stage and higher histological grade which might manifest poor outcome.

Several genes in our model had been studied in human cancers. CBLC (CBL proto-oncogene c), a homologue of CBL protein family, which could activate receptor tyrosine kinases (RTK), was frequently elevated in non-small cell lung cancer (NSCLC), but the function of CBLC in tumorigenesis remained still unknown [19]. PSMC4 (proteasome 26S subunit, ATPase, 4), was recognized as a house keeper gene in breast cancer [20], but was detected upregulated in prostate carcinoma, which might promote tumorigenesis [21]. Ye et al. [22] discovered that decreased PDIA3 (protein disulfide isomerase family A, member 3, also known as ERp57) expression could enhanced apoptosis, and suppressed proliferation, invasion, and migration of acute myeloid leukemia cells. Fibroblast growth factor 8 (FGF8) was recognized as an oncogene, and elevated gene expression in hormonal cancers such as prostate cancer and breast cancer, was associated with a poor prognosis [23]. Chang et al. [24] sequenced 10 samples and found out that oncogene PDGFRA (platelet-derived growth factor receptor, alpha polypeptide) was mutated in endometrial cancer. Similarly, mutations in PDGFRA and/or KIT were found in 5 endometrial carcinosarcomas (5/34, 14.7%) [25]. ESRRA (estrogen-related receptor alpha), which shared structural similarities with estrogen receptors, was reported to play a role in endometrial cancer tumorigenesis by Yoriki et al. [26]. Recently, they discovered that ESRRA could be a target of TGF-β to promote epithelial–mesenchymal transition in endometrial cancer. Positive correlations between Corticotropin releasing hormone receptor 1 (CRHR-1) and PR expression might be associated with more advanced FIGO stage disease in Miceli et al’ s findings [27]. Otherwise, Graziani et al. [28] revealed that CRH induced time- and concentration-dependent inhibition of Ishikawa cell growth through the cAMP-PKA pathway. Niwa et al. [29] detected Lymphotoxin-alpha (LTA) C804A and A252G polymorphisms in 110 endometrial cancer patients. They found out either one or two of the variant alleles was associated with a significantly lower risk of endometrial cancer (OR = 0.54, 95% CI 0.33–0.87, P = 0.012). Of the 14 genes, five genes as Serotonin (5-HT) receptors 3E subunits (HTR3E), TRAV30, SBDS, NR2F1 and TNFRSF18 were firstly reported in endometrial cancer.

Similar to our research, Liu et al. [30] constructed a panel of 7 DEG signatures consisting of PHLDA2, GGH, ESPL1, FAM184A, KIAA1644, ESPL1, and TRPM4 to predict the prognosis of endometrial cancer. Wang et al. [31] identified a six-gene model consisting of CTSW, PCSK4, LRRC8D, TNFRSF18, IHH, and CDKN2A by using robust likelihood‐based survival modeling for endometrial cancer. Besides, a lncRNA signature comprising LINC00491, LINC00483, ADARB2-AS1, and C8orf49 showed remarkable prognostic value in endometrial cancer [32].

Unlike previous researches, we firstly focused on the differentially expressed immune-related genes and set up and validated a novel immune-related signature for prognostic model. Tumor microenvironment in endometrial cancer had been elucidated by Sahoo et al. [33]. The dynamic interaction between tumor cells and microenvironment was essential for tumor proliferation, progression and migration. For instance, stromal estrogen receptor (ERα) mediated the mitogenic effects of estrogen on endometrial cell proliferation [34], the same to the gene ESRRA in this model. In our findings, we figured out several immune genes that might be correlated to tumor microenvironment and could provide guidance for the research on tumor microenvironment and tumor pathogenesis.

However, there were some limitations in our research. Firstly, the sample size in our study was small and a larger cohort and more abundant sequencing results were needed. Secondly, we only focused on the gene expression level, but ignored other events such as the gene mutation, methylation, and copy number amplification, which were also important in tumor progression.

## Conclusions

In conclusion, we constructed and validated the prognostic value of a new risk score system, which might serve as a potential predictor for endometrial cancer. Our findings revealed that the model was strongly correlated with clinical characteristics and we guessed that high-risk patients might possess advanced stage and low/moderate grade. Furthermore, our data might have potential to guide personalized treatment and explore new biomarkers for targeted therapy in endometrial cancer.

## Supplementary information

**Additional file 1: Table S1.** Primer sequences of 14-genes mRNA for reverse transcriptional PCR.

**Additional file 2: Table S2.** Differentially expressed immune genes between endometrial cancer and normal tissues.

**Additional file 3: Table S3.** Clinicopathological characteristics of 34 patients with endometrial cancer in FUSCC.

**Additional file 4: Figure S1.** The association between clinical variables and each genes in this risk-score system.

## Data Availability

The data was available in The Cancer Genome Atlas database (https://cancergenome.nih.gov/); IMMPORT database (https://www.immport.org/) and the Cistrome Project (https://www.cistrome.org/).

## Abbreviations

UCEC: Uteri corpus endometrial carcinoma
TCGA: The Cancer Genome Atlas
DEGs: Differentially expressed genes
TF: Transcriptional factors
KEGG: Kyoto Encyclopedia of Genes and Genomes
GO: Gene Ontology
PPI: Protein–protein interaction
ROC: Receiver operating characteristic
AUC: The area under the curve
