# Supplementary figures and images for: A novel risk score system of immune genes associated with prognosis in endometrial cancer
Source: Cancer Cell Int. 2020 Jun 15;20:240. doi: 10.1186/s12935-020-01317-5 (PMC7294624; doi:10.1186/s12935-020-01317-5)

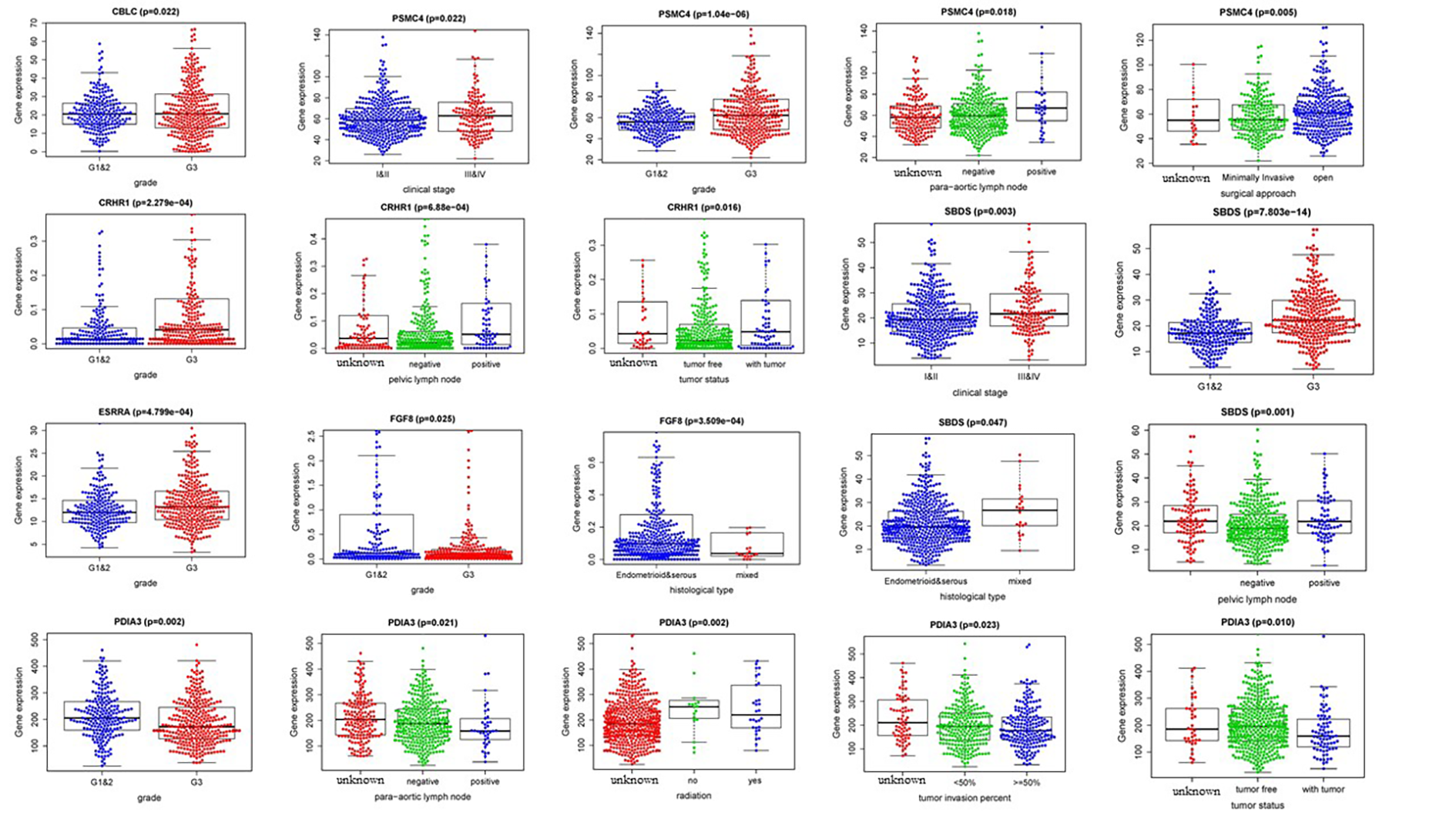

Supplement: Supplementary file 4 — Additional file 4: Figure S1. The association between clinical variables and each genes in this risk-score system. [file 12935_2020_1317_MOESM4_ESM.zip › supplementary figure 1(1).tif]

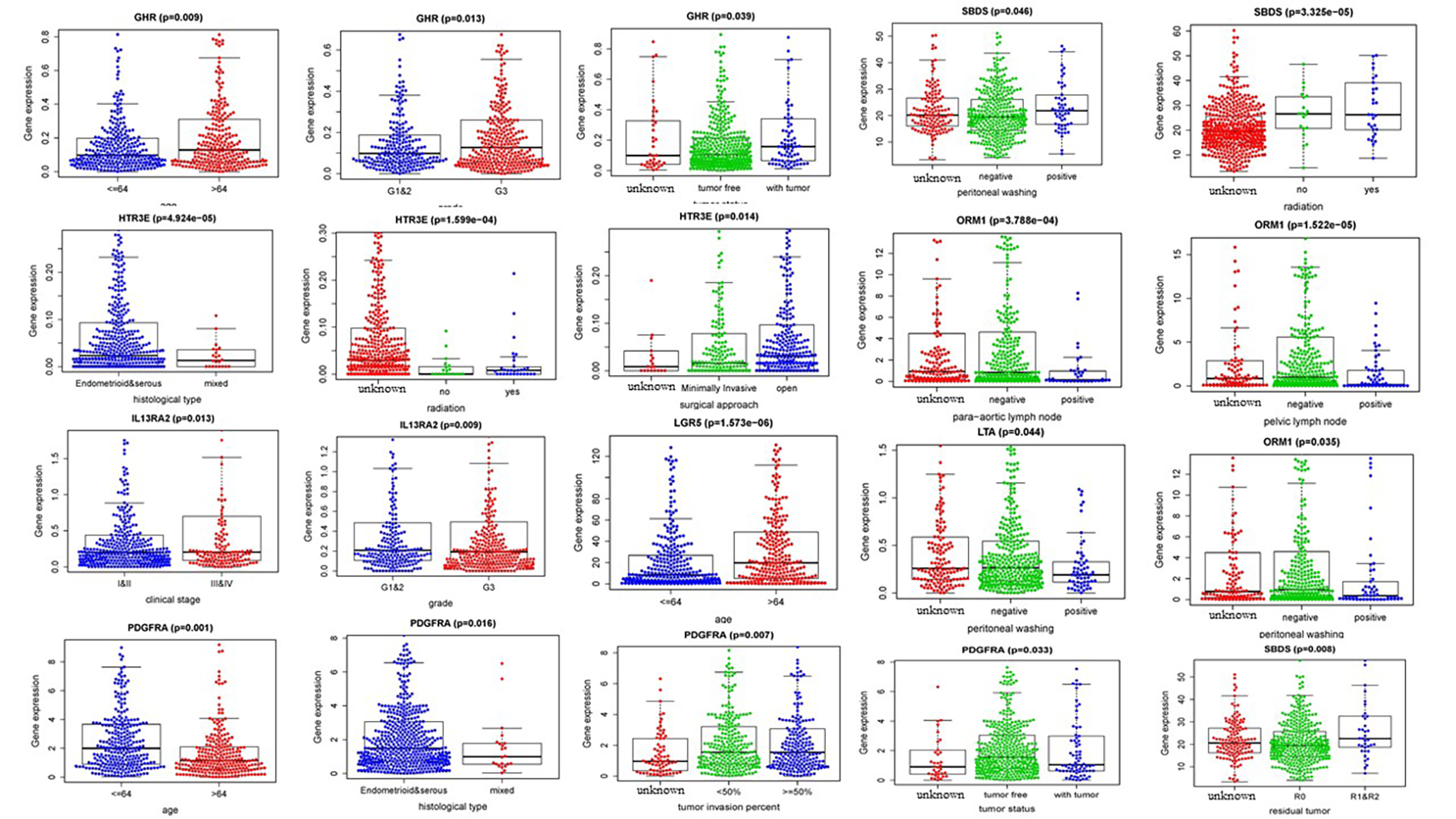

Supplement: Supplementary file 4 — Additional file 4: Figure S1. The association between clinical variables and each genes in this risk-score system. [file 12935_2020_1317_MOESM4_ESM.zip › supplementary figure 1(2).tif]

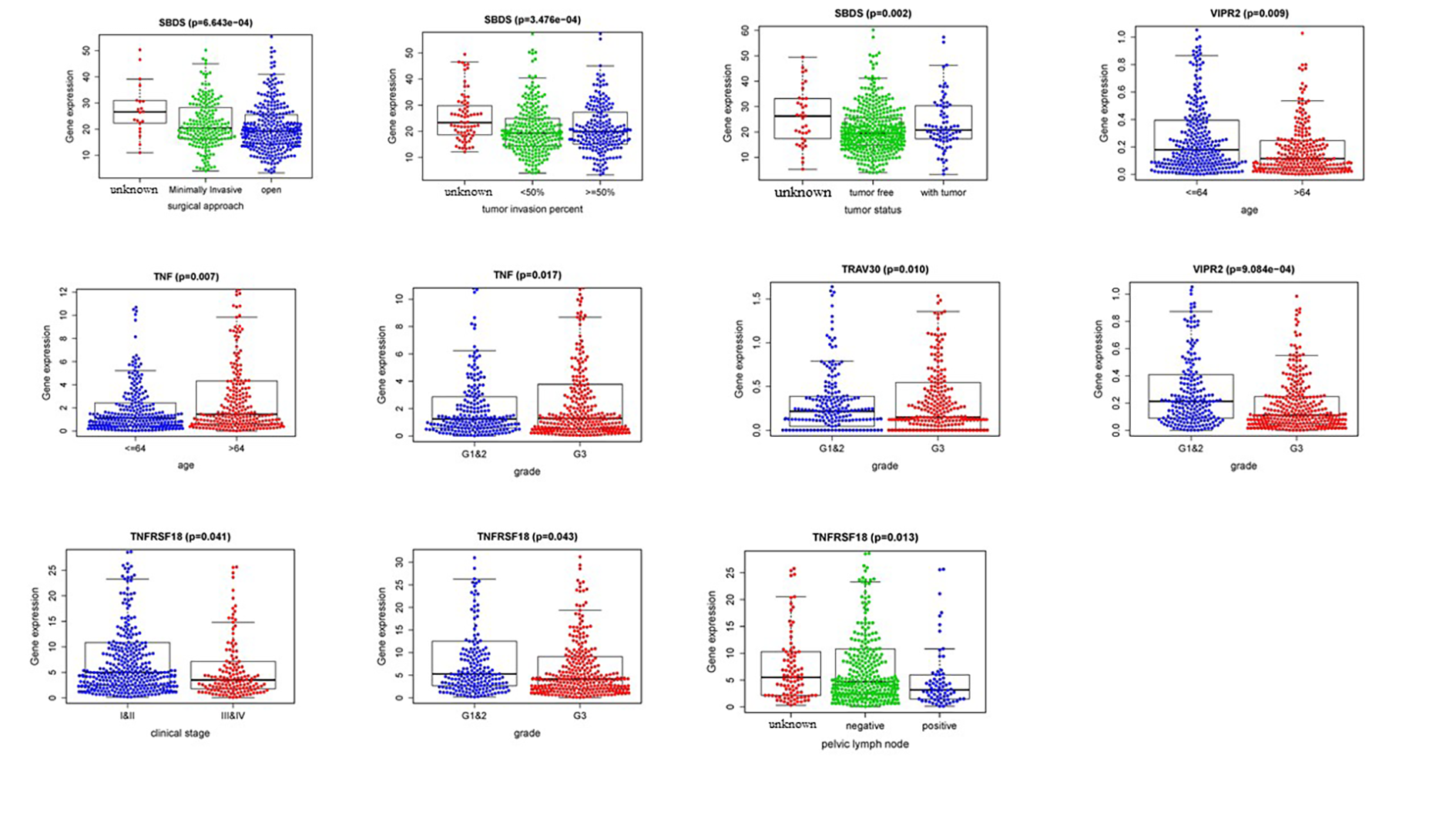

Supplement: Supplementary file 4 — Additional file 4: Figure S1. The association between clinical variables and each genes in this risk-score system. [file 12935_2020_1317_MOESM4_ESM.zip › supplementary figure 1(3).tif]
